# Supplementary material for: Influenza A Virus Infection Reactivates Human Endogenous Retroviruses Associated with Modulation of Antiviral Immunity
Source: Viruses. 2022 Jul 21;14(7):1591. doi: 10.3390/v14071591 (PMC9320126; doi:10.3390/v14071591)
Supplement: Supplementary file 1 [file viruses-14-01591-s001.zip › MotifAnalysis.zip!/MotifOutput_SC35M/homerResults/motif2.similar.html]

motif2

## Information for motif2

A
T
C
G
C
G
T
A
A
C
T
G
C
G
T
A
G
T
A
C
G
T
C
A
A
G
T
C
C
G
A
T
A
C
G
T
A
C
G
T
  
Reverse Opposite:  

C
G
T
A
C
G
T
A
C
G
T
A
A
C
T
G
A
C
G
T
A
C
T
G
A
C
G
T
A
G
T
C
A
C
G
T
A
T
G
C
  

|  |  |
| --- | --- |
| p-value: | 1e-17 |
| log p-value: | -4.112e+01 |
| Information Content per bp: | 1.913 |
| Number of Target Sequences with motif | 13.0 |
| Percentage of Target Sequences with motif | 31.71% |
| Number of Background Sequences with motif | 329.3 |
| Percentage of Background Sequences with motif | 0.70% |
| Average Position of motif in Targets | 80.3 +/- 61.6bp |
| Average Position of motif in Background | 95.5 +/- 75.0bp |
| Strand Bias (log2 ratio + to - strand density) | 1.6 |
| Multiplicity (# of sites on avg that occur together) | 1.23 |
| Motif File: | file (matrix) reverse opposite |

### Similar de novo motifs found

|  |  |  |  |  |  |  |  |
| --- | --- | --- | --- | --- | --- | --- | --- |
| Rank | Match Score | Redundant Motif | P-value | log P-value | % of Targets | % of Background | Motif file |
| 1 | 0.723 | A C G T C T A G A C T G C G T A A C T G C G T A C G T A A G T C A G T C A C G T A C G T A C G T | 1e-14 | -32.591682 | 17.07% | 0.09% | motif file (matrix) |
| 2 | 0.687 | C G T A A C T G C G T A G T C A A G T C A G T C A C G T A C G T | 1e-13 | -30.274088 | 31.71% | 1.64% | motif file (matrix) |
| 3 | 0.640 | C T G A C T G A C G A T A C T G A G T C A T G C C G A T T A G C T C A G C A G T G C A T C G T A | 1e-12 | -28.312063 | 19.51% | 0.30% | motif file (matrix) |
| 4 | 0.613 | A G T C A C G T A C T G A C G T A G T C A C G T A G T C A G T C | 1e-3 | -7.720204 | 14.63% | 2.43% | motif file (matrix) |
